# One Health and Neglected Tropical Diseases—Multisectoral Solutions to Endemic Challenges

**DOI:** 10.3390/tropicalmed6010004

**Published:** 2020-12-29

**Authors:** Jennifer K. Peterson, Jared Bakuza, Claire J. Standley

**Affiliations:** 1 University Honors College, Portland State University, Portland, OR 97207, USA; jenni.peterson@gmail.com; 2 Department of Biological Sciences, Dar es Salaam University College of Education, University of Dar es Salaam, Dar es Salaam, Tanzania; bakuzajared@yahoo.co.uk; 3 Center for Global Health Science and Security, Georgetown University, Washington, DC 20057, USA

One Health is defined as an approach to achieve better health outcomes for humans, animals, and the environment through collaborative and interdisciplinary efforts. Increasingly, the One Health framework is being applied to the management, control, and even elimination of neglected tropical diseases (NTDs). NTDs are a set of debilitating and often chronic infectious diseases that, collectively, affect more than one billion people in almost 150 countries, with disproportionate impact on the extremely poor [1,2]. In this Special Issue, we present a diverse body of work united under the One Health ideology and a desire to mitigate the devastating effects of NTDs. The numerous diseases, methodologies, and landscapes presented highlight the interconnected and increasingly overlapping existence of humans, animals, and their pathogens.

The global scope of the papers demonstrates the scale at which NTDs affect daily life. From Latin America and the Caribbean to South Asia and sub-Saharan Africa, NTDs are an ever-present reality for far too many people. The articles also highlight that NTDs are both an urban and a rural problem, and the frequency with which they cause disease in animals and persist in zoonotic reservoirs exemplifies the expansive utility of the One Health approach across diverse disease systems. 

Broadly, the works presented touch upon three common themes. First, the nature of zoonotic NTDs is such that eradication is rarely an option; animal reservoirs provide a persistent source of re-infection, leaving local elimination in human populations as the most feasible public health goal. One Health coordination across multiple sectors and disciplines is therefore required to achieve such goals, which necessitates interrupting ongoing transmission, diagnosing and treating current cases in both humans and animals, and preventing future transmission scenarios from re-emerging via human practices that facilitate animal-human contact and/or human-vector contact. The “how” is of course much more complex, and it varies by disease. For example, in Zambia, Mulenga et al. [3] describe that, while tsetse fly management efforts have focused attention on areas of high livestock production, the lack of integration with human or wildlife considerations has resulted in decreased surveillance of wildlife protection areas and game parks, despite potential risk to local communities and international visitors. In some contexts, ethical considerations may also be involved. For example, in their review of rabies as a historical public health concern in India, Radhakrishnan and colleagues [4] note conflicting perspectives on the importance of animal welfare with respect to management of feral dogs, a major source of human rabies in the country. Finally, the work presented in Boyce et al. [5] demonstrates how a lack of holistic understanding about transmission pathways can complicate control or prevention of a disease. In Chad, increasing numbers of Guinea worm disease cases detected in dogs pose a major obstacle for elimination of the disease in humans, although the exact pathways by which dogs are exposed and/or contribute to onward transmission is still under debate. 

Second, some of the works in this issue touch upon bureaucratic or semantic obstacles standing in the way of countries and organizations receiving international support for combatting certain diseases. Support from global health agencies such as the World Health Organization (WHO) is often critical for disease control, but to receive such assistance, the country or disease in question must undergo what can often, regrettably, be a political process to receive formal designation or recognition that a disease is “endemic”, “neglected”, or “zoonotic”. Such is the case with Chagas disease in Trinidad and Tobago, where despite mounting evidence that *Trypanosoma cruzi* transmission to humans occurs , the nation is not yet formally recognized as ‘Chagas endemic’ by WHO [6], and as such, does not receive support to combat the disease. Similarly, toxocariasis and giardiasis are not officially recognized as NTDs by WHO, despite having many of the same characteristics and impacts, not to mention the potential benefits for increased scientific attention, funding, and prioritization that could come with being grouped with the NTDs [7,8]. Finally, as mentioned above, existing eradication approaches for Guinea worm disease have been focused solely on humans; recognition of its zoonotic nature is required to ensure future success [5]. We hope that these studies encourage us to re-examine the power wielded by semantics in public health, and also to think about how we might loosen the grip that official designations such as “NTD”, “endemic”, and “zoonotic” have on global health resource allocation. These words are meant to facilitate and direct global health efforts, but, sadly, in these cases, may function as exclusionary devices.

Finally, several of the articles in this issue provide exciting opportunities for integrating new strategic and methodological approaches to enhance One Health approaches. For instance, Chowdhury et al. [9] focus on improving point-of-need diagnostics with new molecular methods for rapid detection of kala-azar leishmaniasis in resource-limited settings. Yeh et al. [10] argue that genomic technologies may be able to track changes in distribution of NTDs such as schistosomiasis in the face of climate change, as well as elucidate shifting parasite-vector dynamics. “Mainstreaming” and “integration” are key buzzwords in NTD control, recognizing the challenges related to sustainable control of these endemic and persistent pathogens in the decades ahead. As Archer et al. [8] highlight, there may be strong scientific rationales for aligning efforts between waterborne parasitic infections such as schistosomiasis and giardiasis, especially when there are substantial knowledge gaps surrounding the distribution, burden, and zoonotic potential of the latter. 

To this end, the articles in this Special Issue present a compelling and comprehensive snapshot of the myriad challenges facing NTD control, highlighting the complex ways in which different pathogens impact—and are impacted by—human, animal and environmental health. Importantly, they also showcase opportunities and innovations for harnessing the collaborative and holistic potential of One Health to reduce the impact of some of the world’s most widespread and burdensome diseases. 
